# Transmission of Zika Virus — Haiti, October 12, 2015–September 10, 2016

**DOI:** 10.15585/mmwr.mm6606a4

**Published:** 2017-02-17

**Authors:** Ito Journel, Lesly L. Andrécy, Dudley Metellus, Jean S. Pierre, Rose Murka Faublas, Stanley Juin, Amber M. Dismer, David L. Fitter, Daniel Neptune, Marie José Laraque, Salomon Corvil, Manise Pierre, Josiane Buteau, Donald Lafontant, Roopal Patel, Jean Frantz Lemoine, David W. Lowrance, Macarthur Charles, Jacques Boncy, Paul Adrien

**Affiliations:** ^1^National Laboratory of Public Health, Port-au-Prince, Haiti; ^2^Directorate of Epidemiology, Laboratory and Research, Port-au-Prince, Haiti; ^3^CDC, Port-au-Prince, Haiti; ^4^Divison of Global Health Protection, CDC; ^5^National Malaria Control Program, Port-au-Prince, Haiti; ^6^CDC, Dar es Salaam, Tanzania.

Zika virus disease is caused by infection with a flavivirus with broad geographic distribution and is most frequently transmitted by the bite of an infected mosquito. The disease was first identified in the World Health Organization’s Region of the Americas in 2015 and was followed by a surge in reported cases of congenital microcephaly in Brazil; Zika virus disease rapidly spread to the rest of the region and the Caribbean (*1*), including Haiti. Infection with the virus is associated with adverse fetal outcomes (*1*) and rare neurologic complications in adults. The magnitude of public health issues associated with Zika virus led the World Health Organization to declare the Zika virus outbreak a Public Health Emergency of International Concern on February 1, 2016 (*2*). Because many persons with mild Zika virus disease are asymptomatic and might not seek care, it is difficult to estimate the actual incidence of Zika virus infection. During October 12, 2015–September 10, 2016, the Haitian Ministry of Public Health and Population (Ministère de la Santé Publique et de la Population [MSPP]) detected 3,036 suspected cases of Zika virus infection in the general population, 22 suspected cases of Zika virus disease among pregnant women, 13 suspected cases of Guillain-Barré syndrome (GBS), and 29 suspected cases of Zika-associated congenital microcephaly. Nineteen (0.6%) patients with suspected Zika virus disease, residing in Ouest (10 patients), Artibonite (six), and Centre (three) administrative departments,* have been confirmed by laboratory testing, including two among pregnant women and 17 in the general population. Ongoing laboratory-enhanced surveillance to monitor Zika virus disease in Haiti is important to understanding the outbreak and ensuring effective response activities.

Haiti’s MSPP first received reports of patients suspected to have Zika virus disease on October 13, 2015, from the Sud Department. MSPP’s Directorate of Epidemiology, Laboratory, and Research (DELR) conducted active investigations and collected serum specimens from 19 patients with suspected Zika virus disease during October 2015–January 2016. The National Laboratory^†^ sent these 19 specimens to the Caribbean Public Health Agency (*3*) for Zika virus testing. On January 15, 2016, MSPP reported that five specimens were positive for Zika virus RNA using the Trioplex reverse transcription–polymerase chain reaction (RT-PCR) assay (*3*).

Standard case definitions^§^ were developed by MSPP in September 2015, and a Zika surveillance module was developed in February 2016; these were disseminated to Haiti’s departments and health facilities (*4*). DELR initiated sentinel Zika virus disease surveillance at 357 health facilities in the National Epidemiology Surveillance Network, using trained surveillance officers who reported the number of suspected cases each week, by age and sex. Pregnancy status of females with suspected Zika virus disease began to be reported in March. Immediate notification of suspected cases of microcephaly and GBS was followed by investigations and laboratory testing (*4*). Surveillance officers completed case investigation forms documenting signs and symptoms of Zika virus disease either when collecting laboratory specimens or after disease confirmation. On February 24, 2016, the National Laboratory introduced in-country multiplex dengue, chikungunya, and Zika testing with the Trioplex RT-PCR assay; all viable specimens collected from 1 to 5 days after symptom onset are tested via the Trioplex assay (*5*). Serologic and plaque reduction neutralization testing for Zika virus currently is not available in Haiti. After immediate notification of suspected cases of GBS or congenital microcephaly, blood specimens are collected. Infants and mothers of infants with congenital microcephaly are tested. For pregnant women with symptoms of Zika virus disease, serum and urine specimens collected within 5 days of symptom onset are tested by RT-PCR; specimens collected >5 days after symptom onset are stored for future Zika testing when additional testing technology is available. For symptomatic persons in the general population, only serum specimens are collected 1 to 5 days after symptom onset only if specimens can be delivered within 2 days to the National Laboratory (*5*) using the national Specimen Referral Network.

Epidemiologic data and laboratory testing results for persons with suspected Zika virus disease and GBS in Haiti presented in this report were obtained from this surveillance system. MSPP personnel were interviewed to describe the Zika virus disease module and response.

## Geographic Distribution

Among 3,036 suspected cases of Zika virus disease (including persons meeting the case definition for Zika virus disease, newborns or stillbirths (including one miscarriage) with suspected congenital microcephaly, and cases of GBS), the highest number originated from Ouest (n = 1,064), Nord (583), and Centre (421) departments. Two peaks occurred in 2016 between epidemiologic weeks 5–7 and weeks 21–27 (Figure). Communes^¶^ with the highest suspected Zika virus disease cumulative incidence rates were Plaine du Nord (441 per 100,000 persons), Milot (234), and Fond des Négres (254).

**FIGURE:**
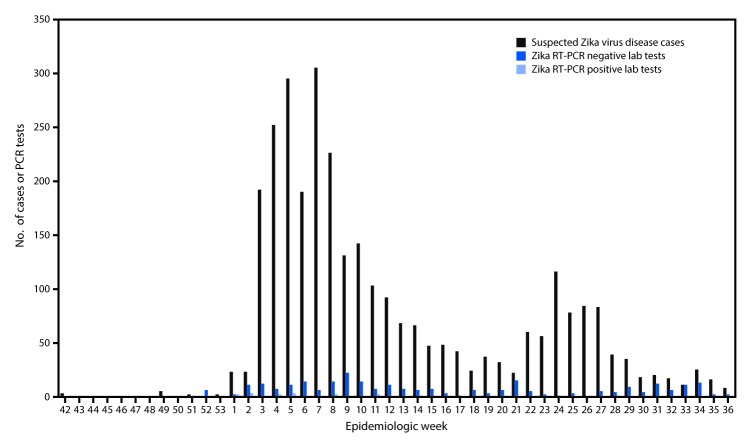
Reported cases of suspected Zika virus disease and RT-PCR testing results*^,^^†^ by epidemiologic week — Haiti, October 12, 2015–September 10, 2016 **Abbreviation:** RT-PCR = reverse transcription polymerase–chain reaction. * Some persons might have had more than one laboratory test. ^†^ A person with a negative Zika RNA test via RT-PCR might have had recent Zika infection detectable by serology and plaque reduction neutralization testing, both of which are unavailable in Haiti.

## Laboratory Testing

Among 294 (9.7%) patients with suspected Zika virus disease, congenital microcephaly, or GBS who underwent testing, 19 cases (6.5%) were confirmed by RT-PCR from serum specimens (five at the Caribbean Public Health Agency and 14 at Haiti’s National Laboratory). Two cerebrospinal fluid specimens and eight urine specimens from other patients tested negative. The median age of patients with confirmed infection was 34 years (range = 0–69 years; interquartile range [IQR] = 12 years) and 44.4% were male. Nine of the confirmed cases were investigated; among these, eight patients reported symptoms including nonpurulent conjunctivitis (seven cases); maculopapular rash (six); temperature >37.2°C (99.0°F) (four); arthritis (three); headache (three); general pain (three); and digestive pain (one). A male fetus (24 weeks’ gestation) had spinal and limb malformations. Blood and urine specimens from the mother were negative, but the umbilical cord blood specimen was positive for Zika virus RNA by RT-PCR.

## Epidemiologic Investigations

DELR completed 148 case investigations; the highest percentage of investigations (90.9%) occurred among pregnant women (Table 1). The most common symptoms reported by patients with suspected Zika virus disease among the general population, pregnant women, and patients with suspected Zika-associated GBS were elevated temperature, headache, and arthralgia (Table 2). Approximately 93% of patients reported mosquitoes in their residences, and 56% reported mosquitoes at work or school. Nine (7.6%) patients reported traveling outside their administrative department in the 2 weeks before symptom onset, indicating potential Zika virus transmission in another department.

**TABLE 1:** Suspected, investigated, and laboratory-confirmed cases of Zika virus disease — Haiti, October 12, 2015–September 10, 2016

Classification	No. of suspected cases	No. investigated (%)	No. RT-PCR–confirmed (%)
Adults/Children	2,972	86 (2.9)	17 (0.6)
Pregnant women	22	20 (90.9)	2 (9.1)
Guillian-Barré syndrome	13	13 (100)	0 (0)
Congenital microcephaly	29	29 (100)	0 (0)
**Total**	**3,036**	**148 (4.8)**	**19 (0.6)**

**Abbreviation:** RT-PCR = reverse transcription–polymerase chain reaction.

**TABLE 2:** Reported signs and symptoms* among investigated cases of suspected Zika virus disease (n = 147) — Haiti, October 12, 2015–September 10, 2016

Symptom/Reported co-infection	Total cases (N = 118)	RT-PCR–confirmed (n = 8)	Adults and children with symptoms of Zika virus disease (n = 85)	Pregnant women (n = 20)	GBS cases (n = 13)
	No. (%)	No. (%)	No. (%)	No. (%)	No. (%)
Temperature >37.2°C (99.0°F)	**89 (75.4)**	4 (50.0)	70 (82.4)	10 (50.0)	9 (69.2)
Headache	**80 (67.8)**	3 (37.5)	64 (75.3)	11 (55.0)	5 (38.5)
Nonpurulent conjunctivitis	**59 (50.0)**	7 (87.5)	49 (57.6)	8 (40.0)	2 (15.4)
Myalgia	**58 (49.2)**	3 (37.5)	51 (60.0)	5 (25.0)	2 (15.4)
Rash	**51 (43.2)**	6 (75.0)	41 (48.2)	9 (45.0)	1 (7.7)
Arthralgia	**64 (54.2)**	3 (37.5)	56 (65.9)	8 (40.0)	3 (23.1)
Digestive symptoms	**28 (23.7)**	1 (12.5)	21 (24.7)	5 (25.0)	2 (15.4)
Other^†^	**26 (22.0)**	2 (25.0)	16 (18.8)	7 (35.0)	0 (0)

**Abbreviations:** GBS = Guillain-Barré syndrome; RT-PCR = reverse transcription–polymerase chain reaction.

* Excludes the 29 cases of congenital microcephaly and one fetus in the adults and children category.

^†^ Tingling (six patients); sore throat (four); itching (three) fatigue (four); edema (two); abdominal pain (two); hypertension (one); chikungunya infection (one); lymphadenopathy (one); and paraplegia (one).

## Congenital Microcephaly, Zika Virus Infection of Pregnant Women, and Guillain-Barré Syndrome

Twenty-nine suspected cases of Zika virus–associated congenital microcephaly (*6*) were detected at 16 health facilities; six infants (20.7%) were delivered in the community. Among the 29 infants with microcephaly, 16 (55%) were female, 26 (90%) were alive at birth, two (3%) were stillborn, and status was not recorded for one. The median number of days from birth until detection of microcephaly was 9 days (range = 0–56 days; IQR = 17 days). Eight live born infants had head circumference measurements at birth, and nine had head circumference measurements 24 hours after birth. Serum specimens were collected for 19 (73%) living infants; all tested negative by RT-PCR. Among 29 infants with microcephaly, 26 (90%) mothers were available for interview. In total, 19 (73%) interviewed mothers reported Zika virus disease symptoms during pregnancy; 14 (54%) reported elevated temperatures, and four (15%) each reported myalgia, rash, and nonpurulent conjunctivitis. The median age of the 26 mothers at delivery was 31 years (range = 16–43 years; IQR = 10 years). The median gestational age at delivery among 13 women for whom this information was available was 37 weeks (range = 18–42 weeks; IQR = 9 weeks).

Among the 22 suspected cases of Zika virus disease identified among women during their pregnancy, field investigations were completed for 20, and 18 women consented to testing. The median age of these women was 32 years (range = 18–39 years; IQR = 6.5 years). On February 24, 2016, two of the 18 women were confirmed to be infected with Zika virus by RT-PCR; they reported rashes, nonpurulent conjunctivitis, and mosquitoes present at home and work.

Thirteen suspected cases of Zika-associated GBS were detected, and 11 patients were hospitalized. The median age was 31 years (range = 2–61 years; IQR = 30 years). Zika-related symptoms included elevated temperature (nine patients) and headache (five). Seven of the 13 GBS cases occurred in females. Among 11 serum specimens submitted to the National Laboratory for testing, two were rejected as inadequate on arrival at the lab; the remaining nine tested negative by RT-PCR. Specimens from these patients might have tested positive for Zika virus by serology and plaque reduction neutralization test, both testing methods that are unavailable in Haiti.

## Public Health Response

MSPP implemented a response plan that included epidemiologic monitoring of Zika virus disease and complications, laboratory testing, vector control, social mobilization, and clinical care. MSPP released statements about modes of transmission, testing, strategies to prevent mosquito bites, and potential Zika virus disease complications through their website, fliers, and radio announcements (*7*). Prevention messages included recommending the use of bed nets and DEET repellents, wearing clothing with long sleeves and pants, covering water containers, and maintaining a clean environment (*3*).

The National Malaria Control Program intensified its long-term vector control response targeting mosquitoes responsible for infections of dengue, chikungunya, and Zika viruses and lymphatic filariasis and malaria. Trained personnel applied larvicide to treat 14,280 larval development sites (*8*) and sprayed insecticide via fumigation trucks in 2,833 areas. Door-to-door household inspections were conducted, and 4,404 households were treated (*8*).

DELR rapidly launched a Zika virus disease module, and weekly analysis of microcephaly and GBS cases was instituted several months later. The Pan-American Health Organization and Global Fund financed Zika virus trainings for 60 trainers in August and 126 surveillance officers in September.

## Discussion

The MSPP’s response to the outbreak has been timely, and the rapid implementation of a Zika virus disease module is reflective of the surveillance system’s flexibility; however, Haiti’s total suspected cases and testing are lower than expected given the population size and transmission dynamics predicted by a modeled estimate of 2.9 million Haitians infected with Zika virus disease (*9*). A strike of health care professionals during March–August 2016 at public health facilities limited the ability to detect and respond to the Zika outbreak. Surveillance for Zika virus disease, congenital microcephaly, and GBS requires clinicians to assess patients systematically for Zika and to recommend testing. In-service national clinical trainings have not been implemented; additional financial resources are needed to increase Zika virus disease testing above 1%, prepare the clinical workforce, and secure Zika serology and PRNT testing.

The findings in this report are subject to at least one limitation. Analysis was limited to Zika confirmation by RT-PCR testing on specimens from persons living at sites from which specimens could be delivered to the National Lab within 2 days. Patients tested after 5 days of the onset of symptoms might have tested negative via RT-PCR, but could have tested positive for Zika through serology and plaque reduction neutralization test. In addition, persons living in Haiti’s rural areas are less likely to be tested for Zika. Therefore, the number of cases of Zika virus disease might be underreported.

As awareness, training, and testing capacity increase, providers should continue to report and test for Zika, chikungunya, and dengue as appropriate, using the existing specimen transport network to increase the number of specimens tested. As novel tests are developed, Haiti’s testing capacity expands, and algorithms change, Haiti will need to adjust its response accordingly. The MSPP is committed to Zika virus disease prevention, systematic detection, and response; however, resources for training and reagents are needed, and coordination of activities will need to be maintained.

**Summary**

**What is already known about this topic?**

Zika virus was first reported in the Region of the Americas in mid-2015 in multiple South American and Caribbean countries and territories. Haiti reported its first confirmed case of Zika virus disease in January 2016.

**What is added by this report?**

Haiti’s Ministry of Public Health and Population has established sentinel Zika surveillance and laboratory testing, and has implemented a public health response to Zika. From October 12, 2015, until mid-September 2016, a total of 3,036 cases of suspected Zika virus infection were identified, including 22 suspected cases in pregnant women, 13 suspected cases of Zika virus–associated Guillain-Barré syndrome, and 29 suspected cases of Zika-associated congenital microcephaly. The National Laboratory tested 294 specimens from suspected Zika virus disease patients; 19 (6.5%) were positive by reverse transcription–polymerase chain reaction testing.

**What are the implications for public health practice?**

Improving reporting of cases of suspected Zika virus disease to public health authorities by health care workers in Haiti and by providers evaluating patients with recent travel to Haiti is important to ongoing surveillance initiatives. Trained epidemiologists and health care workers are important in ensuring reporting. Although laboratory-confirmed Zika virus disease cases were detected in only three of Haiti’s 10 departments (geographic administrative units), testing should continue throughout the country according to Haiti’s guidelines. Integrated mosquito control strategies can mitigate disease spread in Haiti. Residents of and visitors to Haiti should follow recommended precautions to protect against Zika virus infection.

## Footnotes

* Haiti is divided geographically into 10 administrative departments. Each department is further divided into 42 arrondissements.

† The National Public Health Laboratory runs Trioplex reverse transcription–polymerase chain reaction tests. All Zika testing reported from October 2015 to September 2016 was done using Trioplex RT-PCR testing.

§ A suspected Zika virus disease case was defined as the occurrence of a temperature >37.2°C (99.0°F) or a rash with one of the following symptoms: headache/discomfort, nonpurulent conjunctivitis (hyperemia), joint pain (arthralgia), or myalgia in any person (excluding newborns). Congenital microcephaly was defined as a head circumference measuring less than the 3rd percentile for gestational age and sex at birth after 24 hours; any newborn with microcephaly is considered to be a suspected case of congenital microcephaly Zika and is investigated. Suspected cases of GBS are defined as symmetric, progressive flaccid paralysis with or without pain or paresthesia, or clinician-diagnosed Guillain-Barré syndrome in a person of any age. A confirmed Zika virus disease case is any suspected Zika case with a positive RT-PCR result or confirmation of Zika by plaque reduction neutralization testing.

¶ Communes (n = 145) make up Haiti’s third geographic administrative unit within arrondissements.
